# Adult-Onset Pilocytic Astrocytoma Predilecting Temporal Lobe: A Brief Review

**DOI:** 10.3390/life12070931

**Published:** 2022-06-21

**Authors:** Nazmin Ahmed, Gianluca Ferini, Kanak Kanti Barua, Rathin Halder, Sudip Barua, Stefano Priola, Ottavio Tomasi, Giuseppe Emmanuele Umana, Nathan A. Shlobin, Gianluca Scalia, Kanwaljeet Garg, Bipin Chaurasia

**Affiliations:** 1 Department of Neurosurgery, Ibrahim Cardiac Hospital and Research Institute, Shahbagh, Dhaka 1000, Bangladesh; nazmin.bsmmu@gmail.com; 2 Department of Radiation Oncology, REM Radioterapia srl, 95029 Viagrande, Italy; gianluca.ferini@grupposamed.com; 3 Department of Neurosurgery, Bangabandhu Sheikh Mujib Medical University, Shahbagh, Dhaka 1000, Bangladesh; drbaruak@gmail.com (K.K.B.); rathin.halder@gmail.com (R.H.); 4 Department of Neurosurgery, National Institute of Neurosciences and Hospital Agargaon, Dhaka 1207, Bangladesh; gnisudipbarua@gmail.com; 5 Division of Neurosurgery, Health Sciences North, Northern Ontario School of Medicine University, Sudbury, ON P3E 2C6, Canada; stefanopriola@gmail.com; 6 Department of Neurosurgery, Christian-Doppler- Klinik, Paracelsus Private Medical University, 5020 Salzburg, Austria; ottavio.tomasi@gmail.com; 7 Department of Neurosurgery, Trauma Center, Gamma Knife Center, Cannizzaro Hospital, 95126 Catania, Italy; 8 Department of Neurological Surgery, Northwestern University Feinberg School of Medicine, Chicago, IL 60611, USA; nathan.shlobin@northwestern.edu; 9 Department of Neurosurgery, ARNAS Garibaldi, 95123 Catania, Italy; gianluca.scalia@outlook.it; 10 Department of Neurosurgery and Gamma Knife, All India Institute of Medical Science, New Delhi 110029, India; drkanwaljeetgarg@gmail.com; 11 Department of Neurosurgery, Neurosurgery Clinic, Birgunj 44300, Nepal; trozexa@gmail.com

**Keywords:** brain tumor, pilocytic astrocytoma, temporal lobe, clinical presentation, outcome

## Abstract

(1) Introduction: Adult-onset pilocytic astrocytoma (APA) accounts for only 1.5% of all brain tumors, and studies regarding APA are limited. This review is focused on the history, clinical course, cytogenetics, neuroimaging features, management, and outcome of APAs. (2) Methods: Using a systematic search protocol in Google Scholar, PubMed, and Science Direct databases, the authors extracted cases of APA predilecting the temporal lobe from inception to December 2020. Articles lacking necessary data were excluded from this study. Data were analyzed using IBM SPSS 23 statistical package software. (3) Results: A total of 32 patients, 14 (43.8%) males and 18 (56.2%) females, with a male/female ratio of 0.77/1, were grouped. The mean age of the patients was 34.22 ± 15.17 years, ranging from 19 to 75. The tumors were predominantly located in the left side. We have also discussed the clinical presentation, and headache was the most common complaint, followed by visual disturbance. Preoperative neuroimaging features demonstrated cystic lesions in 16 patients, with mural nodule in 5 patients; intracerebral hemorrhage was present in 1 patient, and solid enhancing mass was observed in 3 patients. Only our reported case presented as a solid calcified mass. Most of the patients (78.1%) underwent a gross total resection (GTR), only 5 (21.9%) underwent subtotal resection (STR). The outcome and prognosis history were excellent, and no recurrence was observed. (4) Conclusion: Most of the APAs of the temporal lobe follow benign clinical courses, but some patients exhibit aggressive clinical behavior. There was no history of recurrence after treatment at up to 27 years of follow-up.

## 1. Introduction

Pilocytic astrocytomas are slow-growing WHO grade I brain tumors that arise from astrocytes [1]. Adult-onset pilocytic astrocytomas (APAs) are also WHO grade 1 neoplasms, which account for only 1.5% of adult brain tumors. These tumors usually develop in children and can arise anywhere in the central nervous system. Most commonly, they are observed near the cerebellum, hypothalamic region, brainstem, or optic nerve [2]. The factors leading to development of pilocytic astrocytomas are still unknown, though there appears to be a genetic basis. PAs most often occur in people with neurofibromatosis type 1 (NF1), tuberous sclerosis, and Li-Fraumeni syndrome [3,4].

In adults, APAs are very rare and, compared with pediatric patients, a large portion have an aggressive clinical course [5,6]. In the last few years, a great effort has been made by researchers to understand histological features and cytogenetic and molecular markers of these tumors [7,8].

Because of the rarity of APAs in the adult population, their biological behavior, molecular cytogenetics, clinical presentation, neuroimaging features, optimum management strategies, and outcome are restricted to case reports, case series, and a few original articles. In this paper, the authors present a case of APA involving the temporal lobe. The astrocytoma mimicked a calcified meningioma preoperatively, and it was confirmed as APA after histopathological analysis. The atypical imaging features led us to search for similar occurrences in the literature. The authors systematically reviewed all the reported cases, addressing APAs with a specific predilection to the temporal lobe to analyze their regional variation based on molecular cytogenetics, neuroimaging features, management strategies, and outcome.

## 2. Materials and Methods

### 2.1. Study Selection

A systematic review of the literature was conducted in Google Scholar, PubMed, and Science Direct databases using the Mesh terms “pilocytic astrocytoma” AND “temporal lobe”. First, we considered all papers regarding adult-onset PA involving the temporal lobe, which included case reports, case series, and original articles. All the published papers were meticulously reviewed for necessary information. We restricted the screening language to English.

### 2.2. Inclusion Criteria

All the available literature regarding adult-onset PA involving the temporal lobe, with or without extension into surrounding structures, was included and reviewed thoroughly. Inclusion criteria were 1. Age: >18 years; 2. New diagnosis of PA; 3. PA confirmed on histopathology; and 4. Articles containing information about the selected demographics, management as described by extent of resection, and outcome in terms of recurrence.

### 2.3. Exclusion Criteria

Articles describing 1. Pediatric presentation of PA, 2. Location other than temporal lobe, and 3. Lack of information regarding the features mentioned above were excluded from the study.

### 2.4. Data Extraction

**Figure 1:**
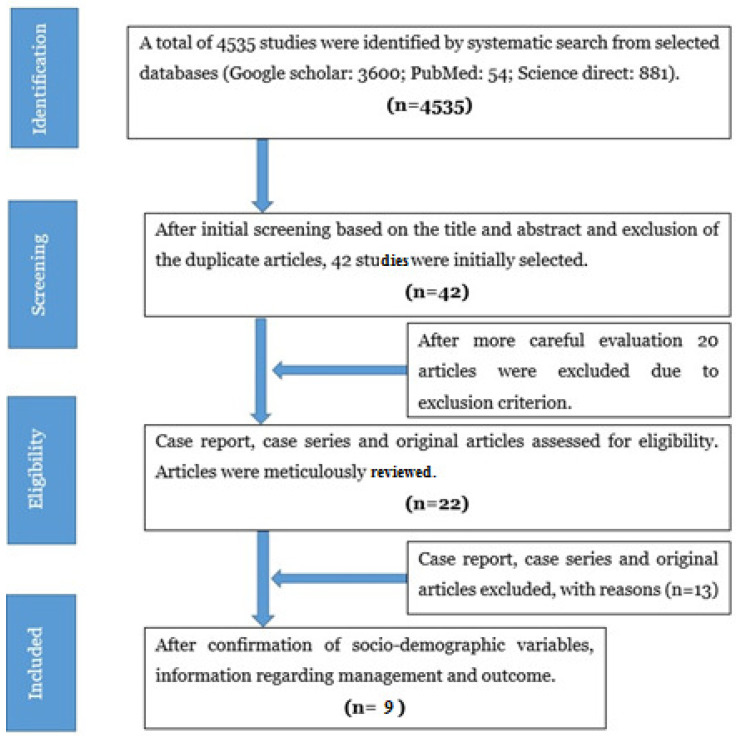
PRISMA flow diagram for study selection.

Using the selected keywords, a systematic search conducted on Google Scholar, PubMed, and Science Direct databases identified 4535 potential articles from inception to December 2020. After careful screening, 42 articles were identified based on the title, abstract, and removal of duplicates. Of those 42 articles, 20 were excluded due to our exclusion criteria. After careful evaluation, 13 more articles were excluded because they lacked socio-demographic variables, information regarding management, and outcome. The study procedure is depicted in a PRISMA [9] flow diagram (Figure 1). Nine papers were considered for systematic review (Table 1).

## 3. Results

The authors selected nine articles for the analysis, with a total of 32 patients, 14 (43.8%) males and 18 (56.2%) females, after the inclusion of the present case. The mean age of presentation is 34.22 (±15.17) years, ranging from 19 to 75. Tumors were predominantly located in the left side. The clinical presentation varied: headache was the most common complaint, followed by visual disturbances. Preoperative neuroimaging features showed a cystic lesion in 16 patients, with mural nodule in 5 patients, intracerebral hematoma in 1 patient, solid enhancing mass in 3 patients. Only our reported case presented as a solid calcified mass. Most of the patients (78.1%) underwent gross total resection (GTR), and five underwent subtotal resection (STR). The prognosis was good, and no recurrence was observed at 6 months—26.5 years of follow-up.

### 3.1. Case Report

#### 3.1.1. History and Examination

A 25-year-old female presented with progressive right-sided headaches for 5 months. She also complained of blurred vision. She had experienced convulsions twice, controlled well with phenytoin for 2 months. On examination, she was conscious, oriented to place and person. The neurological examination revealed left-sided homonymous hemianopia with normal visual acuity and fundoscopic findings. There were no other focal neurological deficits.

#### 3.1.2. Preoperative Imaging

**Figure 2:**
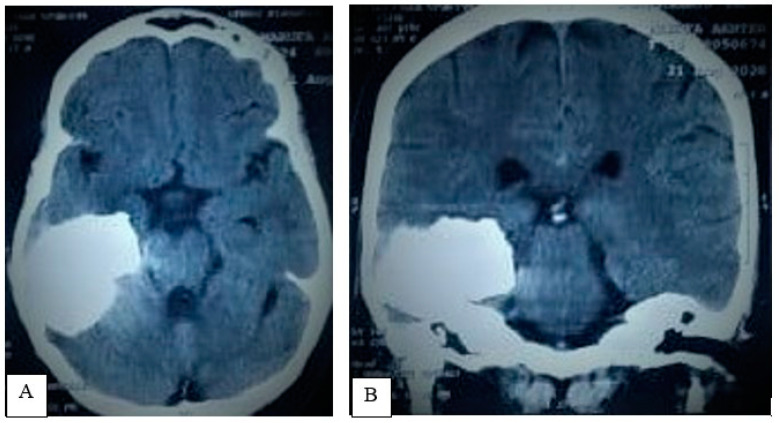
Brain CT scan: axial (**A**) and coronal (**B**) images demonstrate an irregular calcified lesion occupying the right middle temporal fossa, anterior to the petrous part of the temporal bone, with minimum mass effect. These features are consistent with middle skull base calcified meningioma.

**Figure 3:**
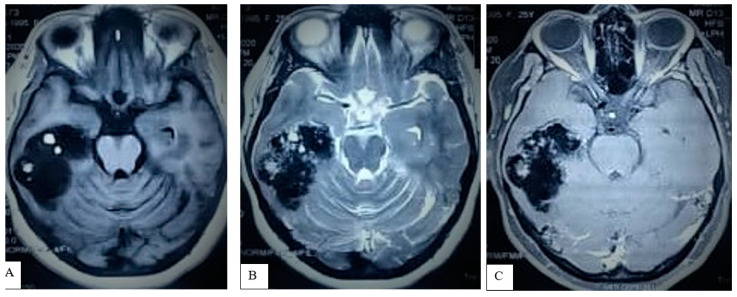
Brain MRI: T1WI (**A**) and T2WI (**B**) axial sections showing a predominantly hypointense lesion with some scattered hyperintense areas, located in the right temporal lobe. The lesion seems to be intra-axial in this sequence. Mass effect is evident by compression on adjacent sulci and gyri with effacement of the right ventricle temporal horn. However, there is no shift of the midline structures. After gadolinium, there is no enhancement (**C**).

A brain CT scan performed at the time of convulsion onset detected a totally calcified lesion (4.6 × 3.6 cm) in the right temporal region, without any other abnormality (Figure 2A,B). A brain MRI with contrast showed a right petrous region mass measuring 4.6 × 3.6 × 3 cm. There was evidence of a dural tail, encasement of the right petrous bone region without contrast uptake by the lesion; these findings radiologically suggested a diagnosis of right middle fossa calcified meningioma, for which the patient was referred for neurosurgical evaluation and definitive management (Figure 3A–C).

#### 3.1.3. Surgical Procedure

**Figure 4:**
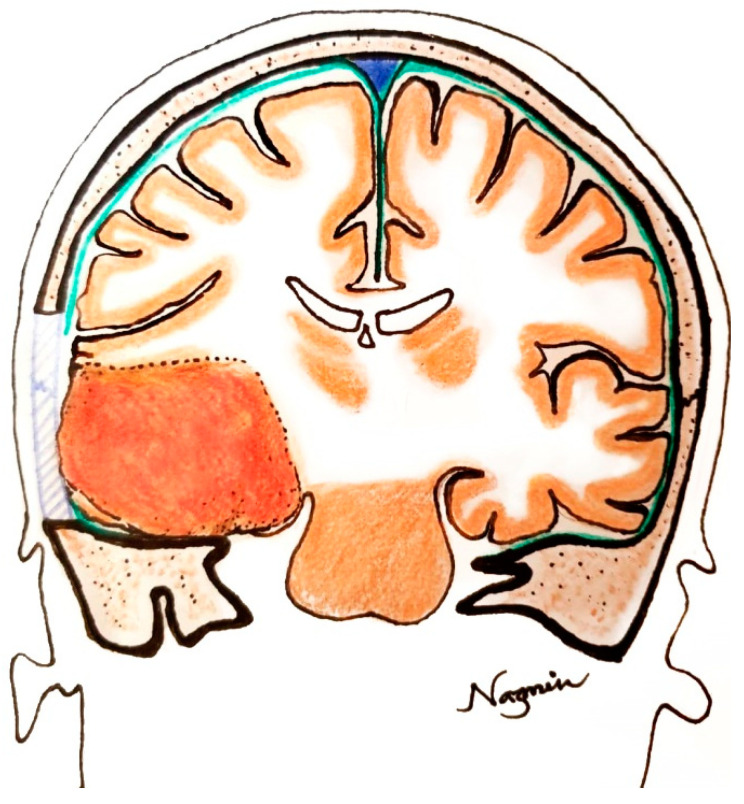
Schematic drawing of the brain: coronal section at the level of the mid-pons demonstrates the topographic relationship of the tumor with the surrounding neurovascular structures in our reported case. The craniotomy area is marked in blue, and durotomy is marked in green.

**Figure 5:**
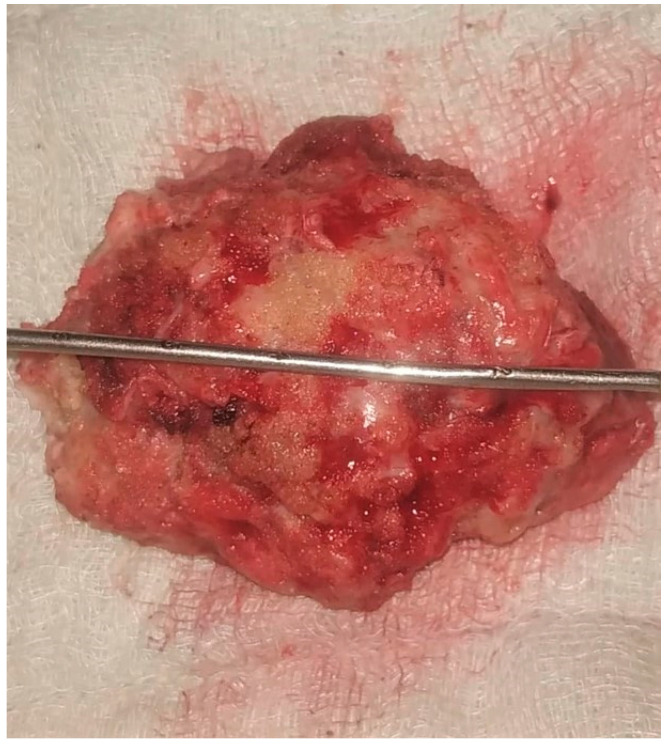
Macroscopic appearance of the tumor, removed en bloc, showing a yellowish-red lesion, measuring approximately 6 × 5 cm, irregular lobulated surface with a hard consistency.

We performed a standard temporal craniotomy and reached the floor of the middle temporal fossa by rongeuring the overhanging bone (Figure 4). The dura was incised in ‘U’ shaped fashion, with the base directed inferiorly, then we performed en bloc removal following the four principles of meningioma surgery (Figure 5). Hemostasis was ensured. Watertight dural closure was obtained, and the wound was closed in layers, leaving no drain tube in situ.

#### 3.1.4. Postoperative Course

The postoperative period was uneventful, with no new neurological deficits.

#### 3.1.5. Histological Examination

Microscopic examination of the resected specimen demonstrated a biphasic appearance of tightly compacted cells with intervening looser areas. There were elongated Rosenthal fibers with eosinophilic proteinaceous inclusions. These features were consistent with the diagnosis of pilocytic astrocytoma.

#### 3.1.6. Follow-Up

There was no evidence of recurrence at the follow-up CT scan after one year.

## 4. Discussion

We provide a case report and systematic review of PAs of the temporal lobe in adult patients. Our study examines the demographics, management, and outcomes of these lesions. In the ensuing discussion, we aim to provide a comprehensive review of the molecular cytogenetics, neuroimaging features, management, and outcome of adult patients with temporal lobe PA.

### 4.1. Molecular Cytogenetics

The genetic events that cause the development of pilocytic astrocytoma are still not well known. Previously conducted studies have reported high chances of occurrence of low-grade glioma in patients with neurofibromatosis type 1 [18]. There are also increased chances of mutations of BRAF, constant chromosome gains at 7q34, and mutations of KRAS activating the MAPK pathway in sporadic pilocytic astrocytoma [19,20,21]. Despite recent advances in the cytogenetics of pilocytic astrocytoma, the molecular blueprint of growth and development is still largely unexplained. Research suggests that the copy-number alterations might play an essential role in PA etiology [22].

### 4.2. Presentation and Neuroimaging Features

Generally, our study indicates that patients present with headache and/or visual disturbances. MRI is the diagnostic modality of choice. Mixed signal intensity in both T1 and T2 weighted sequences, with marked heterogeneous contrast enhancement, can be observed. Usually, as indicated by our study, they are cystic lesions surrounded by mild peritumoral edema [23].

### 4.3. Recommended Management Strategy

The results of this study emphasize the clinical heterogeneity that can be found in adult patients with PA. Surgery is considered the primary treatment of PA. The main goal should be the complete total macroscopic resection of the tumor in the first surgery attempt [24,25]. If the tumor cannot be resected completely, radiotherapy and chemotherapy should be attempted for the remaining sections. Additionally, these options may be utilized for patients who are not surgical candidates [26].

However, the role of radiotherapy in the postoperative management of remaining tumors is still unclear [27]: some studies show a benefit in survival or tumor control, while others show none [28,29,30]. Although exceedingly rare, cases of malignant transformation in pilocytic astrocytomas have been documented, which must be considered prior to considering adjuvant therapies [31,32]. Similarly, the role of adjuvant chemotherapy is still unclear [33]. Nevertheless, in young patients with a tumor that is inoperable or difficult to remove, chemotherapy can be of some use in delaying radiotherapy, thus preventing unwanted side effects of radiation, such as damage to the developing brain [34,35].

Clinicians must also be cognizant of those likely to be at increased risk for early recurrence and aggressive tumor behavior, that is, patients who have undergone subtotal tumor resections or biopsies, tumors with Ki-67 indices of 5% or more and/or high mitotic rates, and older age at presentation (i.e., >40 years). Patients in the high-risk categories above should be strongly considered for further adjuvant therapy with treatments such as conformal external beam irradiation, stereotactic radiosurgical boost, or chemotherapy [36].

### 4.4. Outcome

The study conducted by Brown et al. confirmed that adults with pilocytic astrocytoma (PA) have a favorable prognosis [37], but other studies contradict these results. A study conducted at the University of Bonn with a series of 44 adult patients reported 10-year progression-free survival and overall survival rates of 67% and 77%, respectively [38]. Another investigation involving 30 adult patients from Princess Margaret Hospital identified and noted 10-year progression-free survival and overall survival rates of 35% and 85%, respectively [36]. A review study of adult PA patients identified in the Surveillance, Epidemiology, and End Results (SEER) Program confirmed younger age and greater extent of resection to be positive prognostic factors [39].

Many studies have shown a good prognosis if the tumor is resected completely. Some have deemed gross total resection (GTR) of APAs to be curative, resulting in superior outcomes when compared with subtotal resection (STR), and therefore GTR or complete resection is strongly recommended for patients with PA [40,41]. However, Ki-67 staining should be performed on all biopsied or resected PA tissue for prognostic purposes and to aid decisions regarding the need for further therapy [42,43].

The location of the tumors is also of great importance in determining the prognosis. Analysis of 865 adult patients with PA from the USA National Cancer Institute (NCI) SEER Program database, using univariate Cox proportional hazards model, revealed a low hazard ratio of death of 0.2 and *p* < 0.0001 for gross total resection compared with subtotal resection or biopsy [39]. Similarly, Stüer et al. found recurrence rates four times higher in patients who underwent partial resection than in those who had a complete resection [24]. No deaths or tumor recurrences were reported in our cohort of patients who have undergone complete resection.

Compared to other locations, pilocytic astrocytomas located in the brainstem have a very poor prognosis, as many structures vital for life lie inside it. Difficult access and incomplete resection are other factors that determine the bad prognosis in this region [35].

## 5. Conclusions

Most of the adult PAs of the temporal lobe follow a benign clinical course, with some patients exhibiting aggressive clinical behavior. There was no history of recurrence after treatment at up to 27 years of follow-up. Despite being classified as a WHO grade 1 neoplasm, PAs may sometimes present as ICH and mimic high-grade lesions in conventional neuroimaging. Maximal safe surgical resection should be the aim of surgery. Molecular markers and Ki-67 leveling index are necessary for targeted therapy to achieve a favorable outcome.

## Figures and Tables

**Table 1:** Reported cases of adult-onset pilocytic astrocytoma of the temporal lobe.

*Case*	*Author*	*Year*	*Age*	*Sex*	*Site*	*Clinical* *Presentation*	*Neuroimaging*	*Management*	*Outcome*
*1*	Garcia and Fulling [10]	1985	24	F	L	HA	Cystic with mural nodule	GTR	No recurrence at 27 years
*2*			27	F	L	HA	Cystic with mural nodule	GTR	No recurrence at 27 years
*3*	Lyons [11]	2007	75	M	L	Aphasia	ICH	GTR	NM
*4*	Li et al. [12]	2008	32	M	R	HA, neck stiffness	Cystic with mural nodule	GTR	No recurrence at 6 months
*5*			34	M	L	HA, visual disturbances	Cystic with mural nodule	GTR	No recurrence at 6 months
*6*	Ellis et al. [13]	2009	24	F	L	Tinnitus	Cystic	GTR	No recurrence at 29 months
*7*			25	M	R	Visual disturbances	Cystic	GTR	No recurrence at 27 months
*8*	Kano et al. [14]	2009	26	F	M	NM	Solid	GTR	No recurrence at 75.5 months
*9*			19	M	M	NM	Solid	STR	No recurrence at 100 months
*10*			32	F	M	NM	Cystic	STR	No recurrence at 18.6 months
*11*	Kitamura et al. [15]	2010	68	M	R	Homonymous quadrantanopia	Cystic with mural nodule	GTR	No recurrence
*12*			72	F	R	HA, nausea, vomiting	Solid enhancing mass	GTR	No recurrence
*13*	Brown et al. [16]	2015	20	M	R	NM	NM	GTR	No recurrence at 24 years
*14*			32	M	L	NM	NM	GTR	No recurrence at 26.5 years
*15*			21	F	L	NM	NM	GTR	No recurrence at 25.8 years
*16*			46	M	L	NM	NM	GTR	No recurrence at 25.1 years
*17*			40	F	L	NM	NM	GTR	No recurrence at 24.5 years
*18*			22	M	R	NM	NM	STR	No recurrence at 21.1 years
*19*			32	F	R	NM	NM	GTR	No recurrence at 14.2 years
*20*			32	F	L	NM	NM	STR	No recurrence at 9.5 years
*21*	Bond et al. [17]	2018	19	M	NM	Seizure	Cystic	GTR	No recurrence at 97 months
*22*			20	F	NM	Seizure	Solid enhancing mass	GTR	No recurrence at 134 months
*23*			24	F	NM	Seizure	Solid enhancing mass	GTR	No recurrence at 26 months
*24*			27	F	NM	Seizure	Cystic	GTR	No recurrence at 22 months
*25*			28	M	NM	Seizure	Cystic	NTR	No recurrence at 65 months
*26*			30	F	NM	Mass effect	Cystic	STR	No recurrence at 116.7 months
*27*			36	F	NM	Seizure	Cystic	Biopsy	No recurrence at 164 months
*28*			40	M	NM	Seizure	Cystic	GTR	No recurrence at 89 months
*29*			41	M	NM	Seizure	Cystic	GTR	No recurrence at 79 months
*30*			42	F	NM	Seizure	Cystic	GTR	No recurrence at 68 months
*31*	Narang et al. [18]	2019	60	F	L	Altered sensorium, speech difficulties	Marginally enhancing mass with ICH	GTR	NM
*32*	Present case	2021	25	F	R	Headache, seizure, visual disturbances	Solid calcified mass	GTR	No recurrence at 6 months

*HA: headache, ICH: intracranial hemorrhage, M: male, F: female, R: right, L: left, NM: not mentioned, GTR: gross total resection, NTR: near-total resection, STR: subtotal resection, RT: radiotherapy.*
